# Presence of CrkI-containing microvesicles in squamous cell carcinomas could have ramifications on tumor biology and cancer therapeutics

**DOI:** 10.1038/s41598-022-08905-7

**Published:** 2022-03-21

**Authors:** Mohamed F. Mohamed, Samer Al-Khudari, Puebla Cassini-Vieira, Amani Erra, Reem Bagabas, Thomas Houser, Kerstin Stenson, Mihir Bhayani, Michael J. Jelinek, Faraz Bishehsari, Timothy M. Kuzel, Sasha H. Shafikhani

**Affiliations:** 1 Division of Hematology/Oncology, Department of Internal Medicine, Rush University Medical Center, Chicago, IL 60612 USA; 2 Department of Otorhinolaryngology, Rush University Medical Center, Chicago, IL 60612 USA; 3 Section of Gastroenterology, Division of Digestive Diseases and Nutrition, Department of Internal Medicine, Rush University Medical Center, Chicago, IL 60612 USA; 4 Cancer Center, Rush University Medical Center, Chicago, IL 60612 USA

**Keywords:** Cancer screening, Head and neck cancer

## Abstract

Recently, we described a phenomenon whereby apoptotic cells generate and release CrkI-containing microvesicles, which stimulate proliferation in surrounding cells upon contact to compensate for their own demise. We termed these microvesicles “ACPSVs” for Apoptotic Compensatory Proliferation Signaling microvesicles. As immune cells and a majority of current cancer therapeutics destroy tumor cells primarily by apoptosis, we conducted a small pilot study to assess the possibility that ACPSVs may also be generated in squamous cell carcinomas. We first evaluated a primary and a metastatic squamous cell carcinoma cancer cell lines for their ability to produce ACPSVs under normal and apoptotic conditions. We next conducted a pilot study to assess the occurrence of ACPSVs in solid tumors extracted from 20 cancer patients with squamous cell carcinomas. Both cancer cell lines produced copious amounts of ACPSVs under apoptotic conditions. Interestingly, the metastatic squamous cell carcinoma cancer cell line also produced high levels of ACPSVs under healthy condition, suggesting that the ability to generate ACPSVs may be hijacked by these cells. Importantly, ACPSVs were also abundant in the solid tumors of all squamous cell carcinoma cancer patients. Detection of ACPSVs in cancer has potentially important ramifications in tumor biology and cancer therapeutics which warrants further investigation.

## Introduction

Squamous cell carcinoma (SCC) is the sixth most common cancer in the developed world^1^. Approximately 66,000 new cases of SCC cancers and ~ 14,500 deaths per year are estimated to occur in the United States alone^2^. Treatment involves surgical resection, radiotherapy, chemotherapy, and immunotherapy, but because of the heterogeneous nature and the complexity of SCC cancers, these highly toxic treatment modalities are often ineffective at reaching a satisfactory long-term outcome, as manifested by the high morbidity and mortality rates associated with these cancers^3^.

We recently reported that under apoptotic conditions, a fraction of epithelial cells with stem-like characteristics, produce and release specialized microvesicles, containing CT10 regulator of kinase I (CrkI), that stimulate proliferation in other cells upon contact^4^. We termed these microvesicles “ACPSVs” for apoptotic compensatory proliferation signaling vesicles. We further demonstrated that ACPSVs were distinct from exosomes and apoptotic bodies, which were also produced by apoptotic cells. We also demonstrated that the ability to stimulate proliferation in neighboring cells was primarily due to ACPSVs, not exosomes or apoptotic bodies. Given that apoptosis is the primary mode of cell death by which immune cells and a majority of cancer therapeutics destroy tumor cells^5–9^, we conducted a pilot study to explore the possibility that ACPSVs may also be generated in SCC tumors and could potentially contribute to tumor persistence, and the disappointing outcomes associated with cancer therapeutics^9,10^.

## Results

### SCC cancer cells produce CrkI-containing ACPSVs under apoptotic conditions

We first evaluated whether SCC cancer cells have the capacity to produce ACPSVs under apoptotic conditions by inducing apoptosis in human tongue squamous cell carcinoma-25 (SCC-25) primary cancer cell line^11^, and the human pharyngeal carcinoma metastatic cancer cell line (Detroit 562)^12,13^, by serum starvation (^4^ and Methods). Forty-eight hours after induction of apoptosis, apoptotic cell death was assessed by probing for apoptotic markers, (caspase-3 and PARP cleavage/processing), and by determining the percentage of cell death, as described^14–19^. Moreover, culture supernatants of apoptotic (serum-starved), and serum-fed non-apoptotic control (healthy) were assessed for their ACPSV contents, by differential centrifugation, probing the 16,000*g* (16K) fractions, which we have shown to contain ACPSVs (^4^ and Methods).

**Figure 1:**
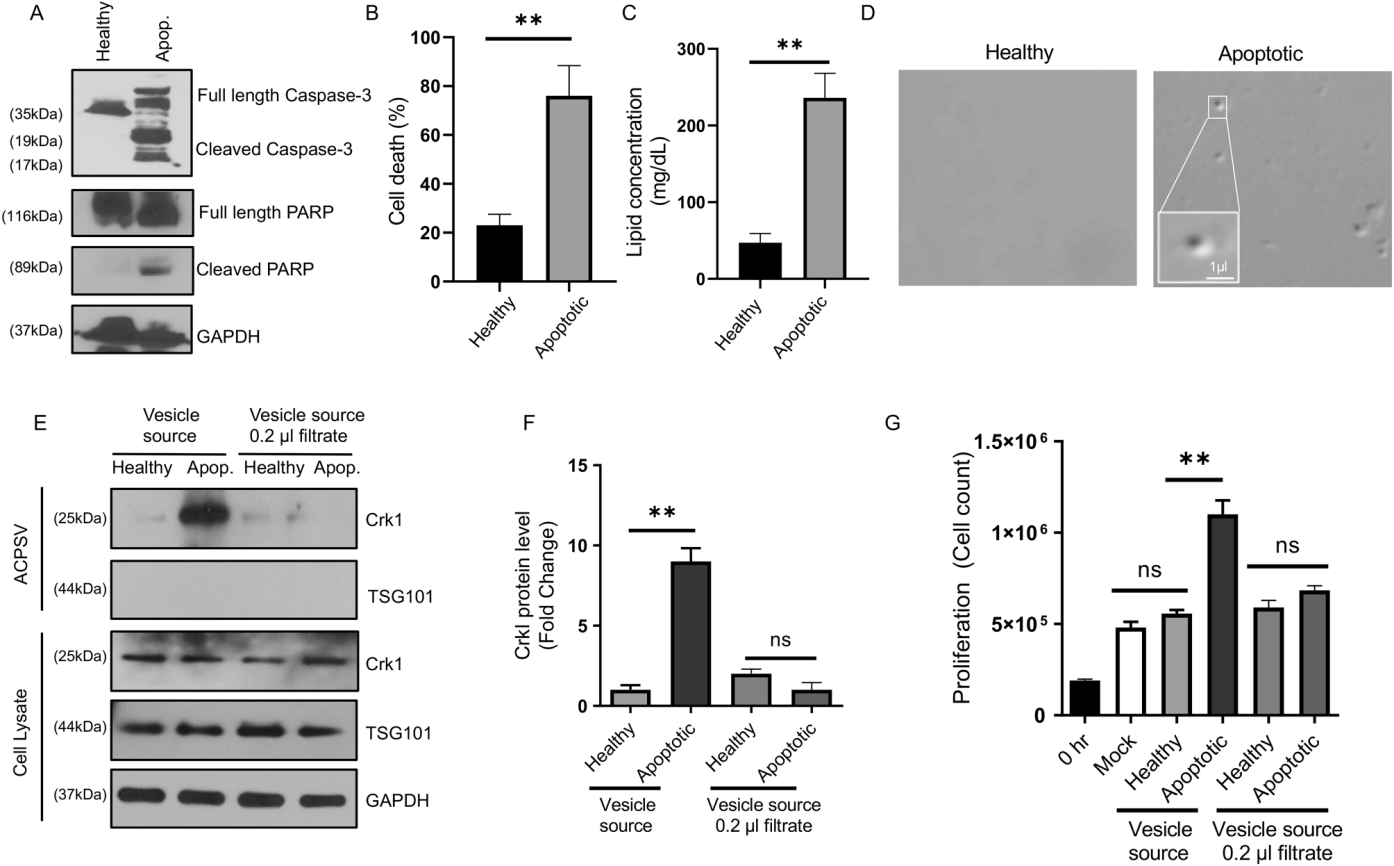
ACPSV production assessment in a primary human SCC cancer cell line. **(A–G)** The human squamous cell carcinoma primary cancer cell line (SSC-25) was either grown in serum-containing media (Healthy) or serum-deficient media (Apoptotic). **(A)** Apoptosis was assessed 48 h after serum starvation by Western blotting for apoptosis markers (activated Caspase-3 and PARP). **(B)** Percent cell death was assessed 48 h after serum starvation. **(C,D)** ACPSVs were purified from culture supernatant of healthy and apoptotic cells and assessed for their ACPSVs contents by lipid concentration assessment **(C)** and visualized by DIC imaging **(D)**. **(E,F)** The 16K fractions from healthy and apoptotic cell cultures were assessed for their ACPSVs by Western blotting, using ACPSV marker (CrkI) before and after passaging through 0.2-micron filters to remove ACPSVs. The 16K fractions were also probed for their exosome contents, using exosome marker TSG101 to show that these fractions are devoid of exosomes. Representative blot images are shown in **(E)** and the tabulated data, shown as the mean ± SEM are shown in **(F)**. **(G)** The 16K fractions of healthy and apoptotic cell cultures (before and after passaging through 0.2-micron filter) were assessed for their ability to stimulate proliferation in adherent HeLa cells. (N = 3; ns, not significant, *p < 0.01, **p < 0.001. Statistical analyses between groups were performed by One-way ANOVA, and pair-wise comparisons within groups were performed or by unpaired Student’s *t*-test).

**Figure 2:**
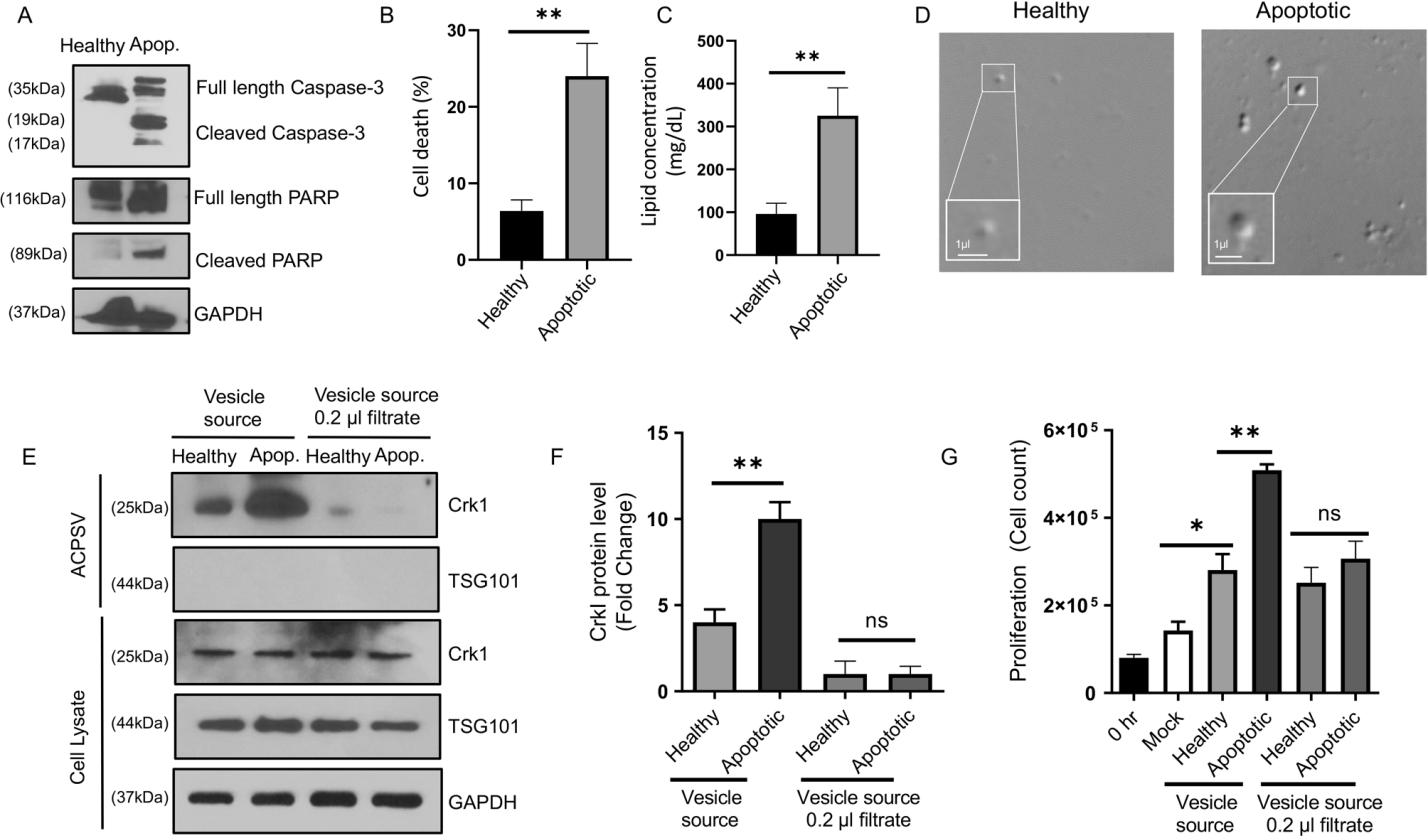
ACPSV production assessment in a metastatic human SCC cancer cell line. **(A–G)** The human pharyngeal carcinoma metastatic cancer cell line (Detroit 562) was either grown in serum-containing media (Healthy) or serum-deficient media (Apoptotic). **(A)** Apoptosis was assessed 48 h after serum starvation by Western blotting for apoptosis markers (activated Caspase-3 and PARP). **(B)** Percent cell death was assessed 48 h after serum starvation. **(C,D)** ACPSVs were purified from culture supernatant of healthy and apoptotic cells and assessed for their ACPSVs contents by lipid concentration assessment **(C)** and visualized by DIC imaging **(D)**. **(E,F)** The 16K fractions from healthy and apoptotic cell cultures were assessed for their ACPSVs by Western blotting, using ACPSV marker (CrkI) before and after passaging through 0.2-micron filters to remove ACPSVs. The 16K fractions were also probed for their exosome contents, using exosome marker TSG101 to show that these fractions are devoid of exosomes. Representative blot images are shown in **(E)** and the tabulated data, shown as the mean ± SEM are shown in **(F)**. **(G)** The 16K fractions of healthy and apoptotic cell cultures (before and after passaging through 0.2-micron filter) were assessed for their ability to stimulate proliferation in adherent HeLa cells. (N = 3; ns, not significant, *p < 0.01, **p < 0.001. Statistical analyses between groups were performed by One-way ANOVA, and pair-wise comparisons within groups were performed or by unpaired Student’s *t*-test).

As expected, serum starvation resulted in increased apoptosis in both cell lines (Figs. 1A,B and 2A,B). Importantly, both cell lines also produced ACPSVs, as assessed by lipid analysis and by differential imaging contrast (DIC) (Figs. 1C,D and 2C,D); and by Western blotting, probing for ACPSV marker (CrkI) (Figs. 1E and 2E). As expected, the aforementioned 16K fractions lacked exosomes, as they probed negative for exosome marker TSG101^20,21^ and had the ability to stimulate proliferation in adherent HeLa cells (Figs. 1G and 2G). Importantly, the 16K fractions from the apoptotic cell cultures were also able to significantly stimulate proliferation, when added exogenously into the medium to adherent cells and removal of ACPSVs in the 16K fractions by passaging these fractions through 0.2-micron filters, abrogated their ability to stimulate proliferation in adherent cells (Figs. 1E–G and 2E–G). Interestingly and in contrast to SCC-25 primary cancer cells which only produced ACPSVs under apoptotic conditions (Fig. 1), the Detroit 562 metastatic cancer cells also produced high levels of ACPSVs, capable of inducing proliferation in adherent cells, even under healthy condition (Fig. 2). These data suggest that these Detroit 562 metastatic cancer cells may have uncoupled the ability to produce ACPSVs from the apoptotic cell death signal that is required to initiate ACPSV production in SCC-25 or in HEK or MEK primary cell lines^4^”.

### CrkI-containing ACPSVs are prevalent in SCC tumors

**Figure 3:**
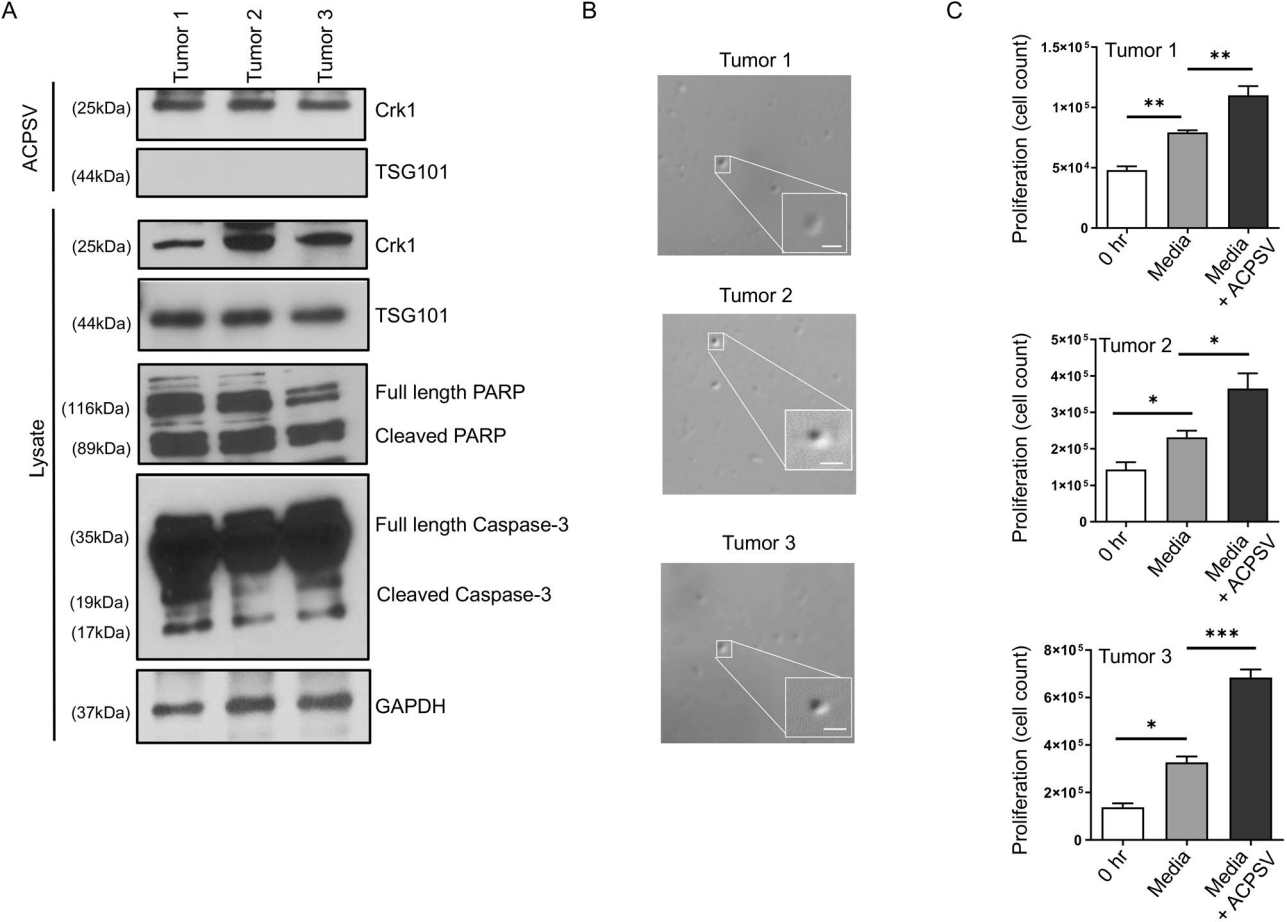
ACPSV production assessment in SCC tumors. **(A)** Resected tumors from 3 SCC cancer patients were assessed by Western blotting for apoptosis (using caspase-3 and PARP activation), and for their ACPSVs contents (after ACPSV purification from the tumors by differential centrifugation) using ACPSV marker CrkI. The 16K fractions (which contain ACPSVs) were also probed for contaminating exosomes (using exosome marker TSG101). **(B)** The ACPSVs in the 16K fractions of these tumors were visualized by DIC. **(C)** The ACPSVs in the 16K fractions of these tumors were assessed for their ability to stimulate proliferation in adherent HeLa cells. **(B)** Purified ACPSVs from these tumors were visualized by DIC imaging. **(A)**; by Western blotting, probing for CrkI **(B)**; and for their ability to stimulate proliferation in adherent HeLa cells by cell count 48 h after treatment with ACPSV-containing media or media alone. (N = 3; ns, not significant, *p < 0.01, **p < 0.001, ***p < 0.0001. Statistical analyses between groups were performed by One-way ANOVA with post hoc test).

We next assessed whether ACPSVs are also generated in SCC human cancers. Toward this objective, we resected tumors from 20 SCC cancer patients (Table 1), as described in the Methods. Demographics included a median age (range) 61 (41–80) with gender, 2 females and 17 males. ACPSVs were prevalent in all tumors after ACPSVs purification from resected specimens (Methods), as evaluated by Western blotting probing for ACPSV marker (CrkI) but not the exosome marker (TSG101); by DIC imaging (Fig. 3B); and by functional analysis assessing for their ability to stimulate proliferation in adherent HeLa cells (Fig. 3C). Figure 3 demonstrates 3 representative SCC subject results with the rest in Table 1. Of note, ACPSVs purified from a resected tumor stimulated proliferation in SCC cancer cell line in a similar fashion to HeLa cells (Fig. S1). Tumors also showed increased apoptosis as determined by Western blotting, probing for apoptotic markers; cleaved caspase-3 and PARP (Fig. 3B).

**Table 1:** CrkI-containing ACPSVs are prevalent in SCC tumors.

Sample #	Tumor				Mets	Adjuvant treatment			ACPSVs (Y/N)*
	Histologic type	Location	Stage	HPV status		Y/N*	Therapy before-after-during sample collection	Radiation before-after-during sample collection	
1	SCC	Larynx	T3N0	NR**	No	Y	Cisplatin—prior	66 Gy. 33fx prior 20 Gy/5 fx—after	Y
2	SCC	Larynx	T4aN0	NR	No	Y	Cisplatin—after	66 Gy/33 fx—after	Y
3	SCC	Oropharynx-tonsil	T2N0	+	No	N	–	–	Y
4	SCC	Larynx	T3N1	NR	No	Y	–	60 Gy/30 fx—after	Y
5	SCC	Oropharynx-tonsil	T2N1	+	Yes—Lung	Y	–	60 Gy/30 fx—after	Y
6	SCC	Oral cavity-tongue	T2N0	NR	No	Y	Keytruda—after	–	Y
7	SCC	Oropharynx-tonsil	T1N2	+	No	Y	Cisplatin—after	66 Gy/33 fx—after	Y
8	SCC	Oropharynx-tonsil	T1N2	+	No	Y	Cisplatin—after	70 Gy/35 fx—after	Y
9	Papillary carcinoma	Thyroid	T1bN1a	NR	No	N	–	–	Y
10	SCC	Oral cavity Tongue	T3N0	NR	No	Y	–	60 Gy/30 fx—After	Y
11	SCC	Parotid lymph node Unknown primary	Unknown primary	NR	Yes	N	–	–	Y
12	Clear Cell Carcinoma	Tongue	T2N0	NR	No	N	n/a	n/a	Y
13	SCC	Parotid	T1N3b	NR	No	Y	n/a	Unknown dose—after	Y
14	SCC	Oropharynx-tonsil	T1N1	+	No	N	n/a	n/a	Y
15	SCC	Oropharynx-base of tongue	T1N1	+	No	N	n/a	n/a	Y
16	SCC	Oral cavity-tongue	T3N0	NR	No	N	n/a	n/a	Y
17	SCC	Larynx	T4aN0	NR	No	Y	n/a	50 Gy/20fx—after	Y
18	SCC	Oral cavity-buccal mucosa	T4N3b	NR	No	N	n/a	n/a	Y
19	SCC	Oral cavity-alveolar ridge	T4aN0	NR	No	N	n/a	n/a	Y
20	SCC	Oral cavity-retromolar trigone	T2N0	NR	No	Y	None	None	Y

*Y (Y = Yes; N = No), **(NR = Not Relevant) n/a = Not Available.

## Discussion

SCC cancers remain a devastating and hard-to-treat set of cancers^3^. Previously, we had reported that cells produce and release CrkI-containing microvesicles (ACPSVs)—(distinct from exosomes and apoptotic bodies)—which stimulate proliferation in healthy neighboring cells upon contact^4^. We further demonstrated that the ability to promote proliferation was primarily due to ACPSVs, not exosomes or apoptotic bodies which were also produced by apoptotic cells^4^. We further demonstrated that ASCPVs stimulated proliferation in healthy neighboring cells by activating c-Jun Kinase (JNK) in the neighboring cells^4^. Given that immune cells and the majority of current cancer cytotoxic therapeutics destroy tumor cells by apoptosis, we assessed the occurrence of ACPSVs in SCC cancer cell lines and their prevalence in SCC human tumors in this report. We show for the first time that ACPS microvesicles are prevalent in all tumors resected from SCC cancer patients. Unfortunately, because of the small tumor size in the samples we received for this study, we were unable to carry out more detailed assessment of ACPSV production in individual tumors in this pilot study. Nevertheless, we believe that this is a strong proof-of-concept study that ACPS vesicles, which we have characterized phenotypically and functionally previously^4^, exist in SCC cancers, building a rationale for future studies to examine their potential roles in tumor proliferation, progression, recurrence, drug persistence/resistance, and the metastatic process. Furthermore, therapeutics directed against these ACPS vesicles may provide a novel strategy for treatment.

Although, there are many factors, impacting tumor persistence and/or resistance to cancer therapeutics^22,23^, one unexplored but potentially important contributing factor to therapy failure in all cancers, including SCC cancers, may be the occurrence of ACPSVs in cancer. ACPSVs could adversely limit drug effectiveness, contributing to the disappointing outcomes associated with therapeutic agents against these cancers. In addition, ACPSVs may also play a role in the metastatic process, due to their ability to potentially disrupt normal tissue structure by stimulating proliferation in normal tissue cells, causing them to dissolve their cellular junctions during the proliferation process, ultimately allowing tumor cells to invade normal tissue. In line with our data that ACPSVs are prevalent in solid tumors and consistent with the notion that ACPSVs may play pivotal roles in tumor biogenesis and metastasis, Crk and JNK have been reported to be overexpressed in tumors, and is associated with tumorigenesis, tumor progression, and poor prognosis with reduced survival rates in many cancers, including oral squamous cell carcinoma^24–29^. Further supporting this hypothesis, mice lacking JNK1 displayed decreased tumor cell proliferation in a mouse model of liver carcinogenesis^30^, and JNK inhibition was found to result in reduction in cell proliferation and capillary tube formation in oral cancer^31^. Interestingly, it has been suggested that oral squamous cell carcinoma (OSCC) cells secrete exosomes, which stimulate proliferation in OSCC cells in a manner that is dependent on JNK activation^32^. Whether it is exosomes or contaminating ACPSVs that drive increased proliferation in OSCC remains to be determined.

Tumor-specific growth rate (TSGR) has been indicated as a temporal prognostic biomarker for the treatment outcomes in patients with non-oropharyngeal squamous cell carcinoma (non-OPSCC) cancers. Patients with high TSGR, (≥ 2.18% per day), have significantly worse overall survival, as compared to those with TSGR below this threshold^33^. However, the factors that influence TSGR remain poorly understood. Primary human and mouse epithelial cells produce ACPSVs only under apoptotic conditions^4^. In contrast, we have found that Detroit 562 metastatic cell line can also produce substantially higher levels of ACPSVs under healthy condition than SCC-25 primary cancer cell line, suggesting that different cancer types may have different capabilities to produce ACPSVs, which could potentially play a pivotal role in their TSGRs. More studies are needed to assess the role of ACPSVs in TSGRs.

We demonstrated that ACPSV biogenesis and production is dependent on CrkI adaptor protein and interfering with CrkI function by mutagenesis or by *Pseudomonas aeruginosa* ExoT (a potent inducer of apoptosis that inactivates CrkI by ADP-ribosylating it^15,16,34^) block ACPSV production without affecting apoptotic cell death^4^. We further showed that interfering with JNK activity also abrogates ACPSV-induced proliferation in neighboring cells. These data indicate that apoptotic cell death and apoptotic compensatory proliferation signaling can be uncoupled from each other. They further suggest that by blocking ACPSV production (i.e., by targeting Crk), or by interfering with ACPSV action (i.e., through the use of JNK inhibitors), we may be able to enhance the effectiveness of current cancer therapeutics.

In summary, we show for the first time that Crk-containing ACPSVs are abundant in squamous cell carcinomas solid tumors, and they potentially could have adverse impacts on tumor biology and cancer therapeutics.

## Methods

All methods in the manuscript were carried out in accordance with relevant guidelines and regulations.

### ACPSV purification from cancer cell lines

Human squamous carcinoma-25 (SCC-25) primary cancer cell line (CRL-1628) and human pharyngeal carcinoma metastatic cancer cell line (Detroit 562, CCL-138) were obtained from ATCC. We induced apoptosis in these cells as described^4^. Briefly, 1 × 10^7^ cells were cultured in 150 mm^2^ flasks (TPP) overnight in 25 mL of indicated media. Next day, cells were induced to undergo intrinsic apoptosis by serum-starvation^35^. 48 h after induction of apoptosis, culture supernatants from serum-fed mock (healthy) and apoptotic cells were collected and subjected to differential centrifugation. In some experiments, the 16K fractions of mock and apoptotic cells were passaged through 0.2-μm filters to remove vesicles in order to show that ACPSVs in these fractions were responsible for the proliferation in adherent cells.

### ACPSV purification protocol

ACPSVs were purified by differential centrifugation as described^4^. Briefly, culture supernatants from serum-fed (Healthy) or serum-starved (apoptosis) were centrifuged at 1500×*g* (1.5 K) for 5 min followed by passage through 5-micron filter (Sterlitech, PES502005) to remove cell debris and apoptotic bodies, followed by centrifugation at 16,000×*g* (16K) for 60 min to collect ACPSVs. ACPSV pellets were resuspended in 300 mL PBS, prior to analyses.

### ACPSV purification from solid tumors

The study protocol was reviewed and approved by the ethics committee and institutional review board (IRB No., 16071102) at Rush University Medical Center. Written informed consent was obtained from each patient. ACPSVs were extracted from solid tumors after surgical resection. Briefly, tumor samples were cut into small pieces by sterile scissors and digested with collagenase D for 30 min at 37 °C. Tissue homogenates were passaged through 70-micron cell strainer then the filtrate were centrifuged at 1500×*g* (1.5 K) for 5 min followed by passage through 5-micron filter to remove cell debris, followed by centrifugation at 16,000×*g* (16K) for 60 min to collect ACPSVs. ACPSV pellets were resuspended in 300 mL PBS, prior to analyses.

### ACPSV characterization and functional assessments

#### Western blotting

Western blotting, as described^19,36^, was performed to evaluate ACPSV production, probing for ACPSV marker, CrkI^4^ and exosome marker (TSG101^20,21^) (Abcam, ab30871). Apoptosis in cells and tumors was detected by probing for apoptotic markers detecting the cleaved caspase-3 (Cell Signaling, 9661), caspase-3 (Cell Signaling, 9662) and PARP (Cell Signaling, 9532). GAPDH (Proteintech, 1094-I-AP) was used as a loading control.

### Lipid analysis

After ACPSV purification, ACPSV levels were estimated by lipid measurement analysis, as described^4^ and according to manufacturer’s guidelines (cell biolabs, Catalog number, STA-613), briefly, 15 µL samples and standards were incubated uncovered at 90 °C for 30 min to completely evaporate organic solvents, after cooling down at 4 °C for 5 min, 150μL of 18 M sulfuric acid were added to each sample, and incubated at 90°c for 10 min. then 100uL of Vanillin Reagent were mixed carefully to each sample. Samples were incubated at 37 °C for 15 min. Samples were read at OD 540 nm by a microplate reader.

### ACPSV functional assessments

5 × 10^4^ HeLa cells/well were seeded overnight in 24-well culture dishes (Costar) in regular DMEM media containing 10% FBS and Penicillin–Streptomycin antibiotics. The next day, the adherent cells were treated with media alone or media containing 50 µl of purified ACPSVs, which were purified from either healthy or apoptotic culture supernatants or from solid tumors. The proliferation-inducing effect associated with ACPSV contents was determined by cell counts after growing recipient cells for 24 h at 37 °C in the presence of 5% CO_2_, using a hemocytometer after trypsin digestion, as described^4^.

### Statistical analyses

Statistical analyses were performed as described previously^4,37–39^. Statistical analyses between groups were performed by One-way analysis of variance (ANOVA) with post hoc testing using Tukey multiple comparison adjustment. Pair-wise comparisons between two groups were performed by unpaired Student’s *t*-test. All analyses were performed using GraphPad Prism software. Statistical significance threshold was set at *p* ≥ 0.05.

### Ethics declarations

The study protocol was reviewed and approved by an independent ethics committee and institutional review board (IRB No. 16071102) at Rush University Medical Center. Written informed consent was obtained from each patient.

## Supplementary Information

Supplementary Figures.

## Data Availability

All data discussed in this manuscript are included in the manuscript.
