# Supplementary material for: Presence of CrkI-containing microvesicles in squamous cell carcinomas could have ramifications on tumor biology and cancer therapeutics
Source: Sci Rep. 2022 Mar 21;12:4803. doi: 10.1038/s41598-022-08905-7 (PMC8938485; doi:10.1038/s41598-022-08905-7)

Supplementary Information

Presence of CrkI-Containing Microvesicles in Squamous Cell Carcinomas Could Have Ramifications on Tumor Biology and Cancer Therapeutics

Mohamed F. Mohamed, PhD [1§], Samer Al-khudari, MD [2,4§], Puebla Cassini-Vieira PhD [1], Amani Erra, MD [1], Reem Bagabas [1], Thomas Houser [2,4], Kerstin Stenson, MD [2,4], Mihir Bhayani [2,4], Michael J. Jelinek[1], Faraz Bishehsari, MD, PhD [1,3,4], Timothy M. Kuzel, MD [1,4], and Sasha H. Shafikhani, PhD [1,4*]

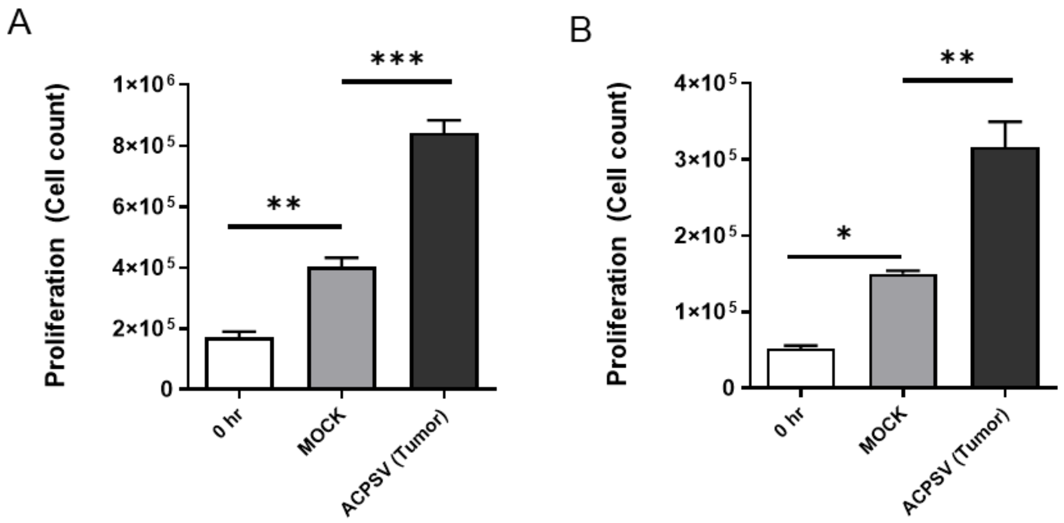

Fig. S1. ACPSV from head and neck tumor stimulate proliferation in the human pharyngeal carcinoma cancer cell line (Detroit 562) similar to HeLa. (A) ACPSVs extracted from a resected tumor from a H&N cancer patient were assessed for their ability to stimulate proliferation in adherent HeLa **(A)** and in adherent Detroit 562 cells **(B).** (N=3; ns, Not significant, *p<0.01, **p<0.001, ***p<0.0001. Statistical analyses between groups were performed by One-way ANOVA with post hoc test).

Uncropped western blots

Figure 1A

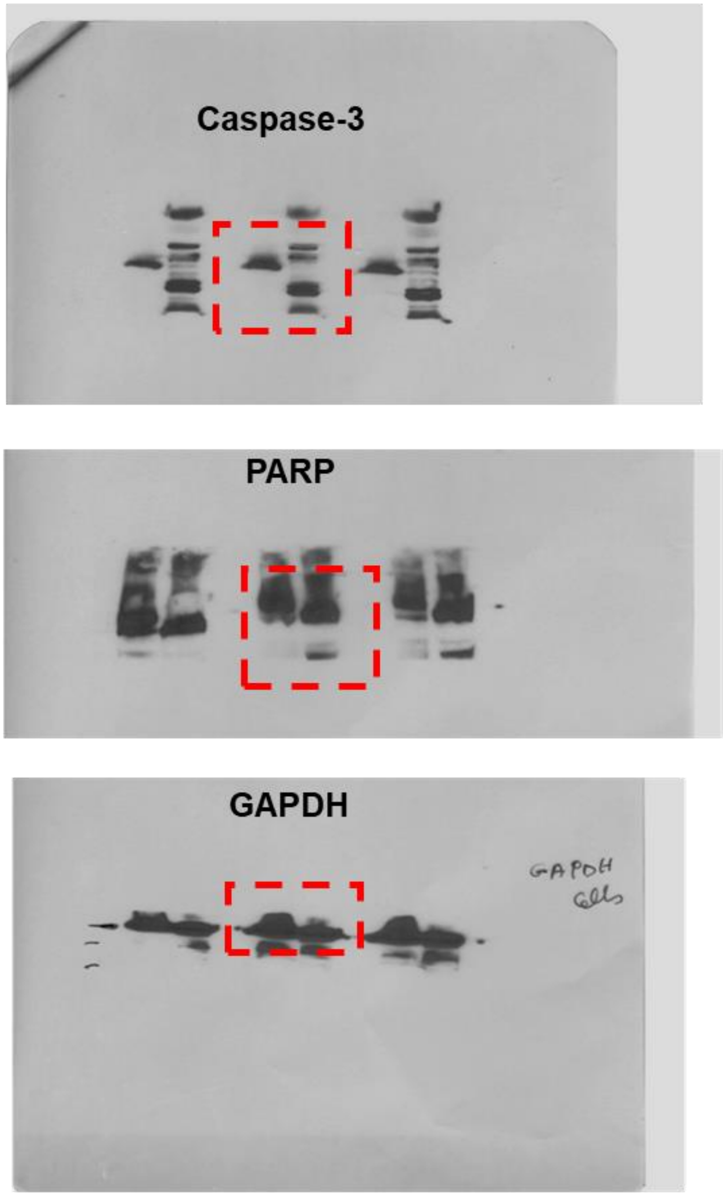

Figure 1E

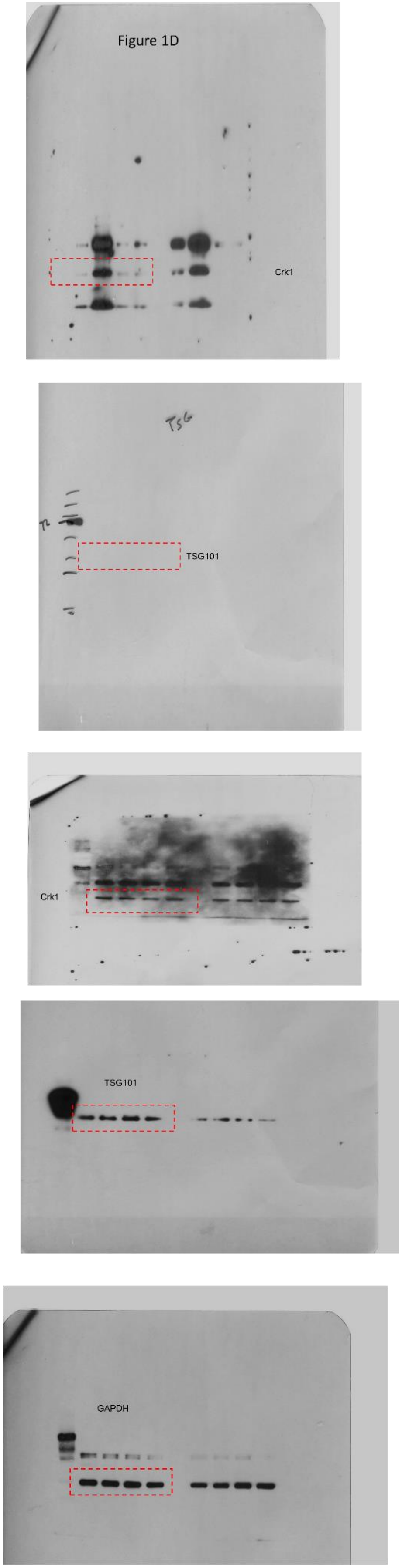

Figure 2A

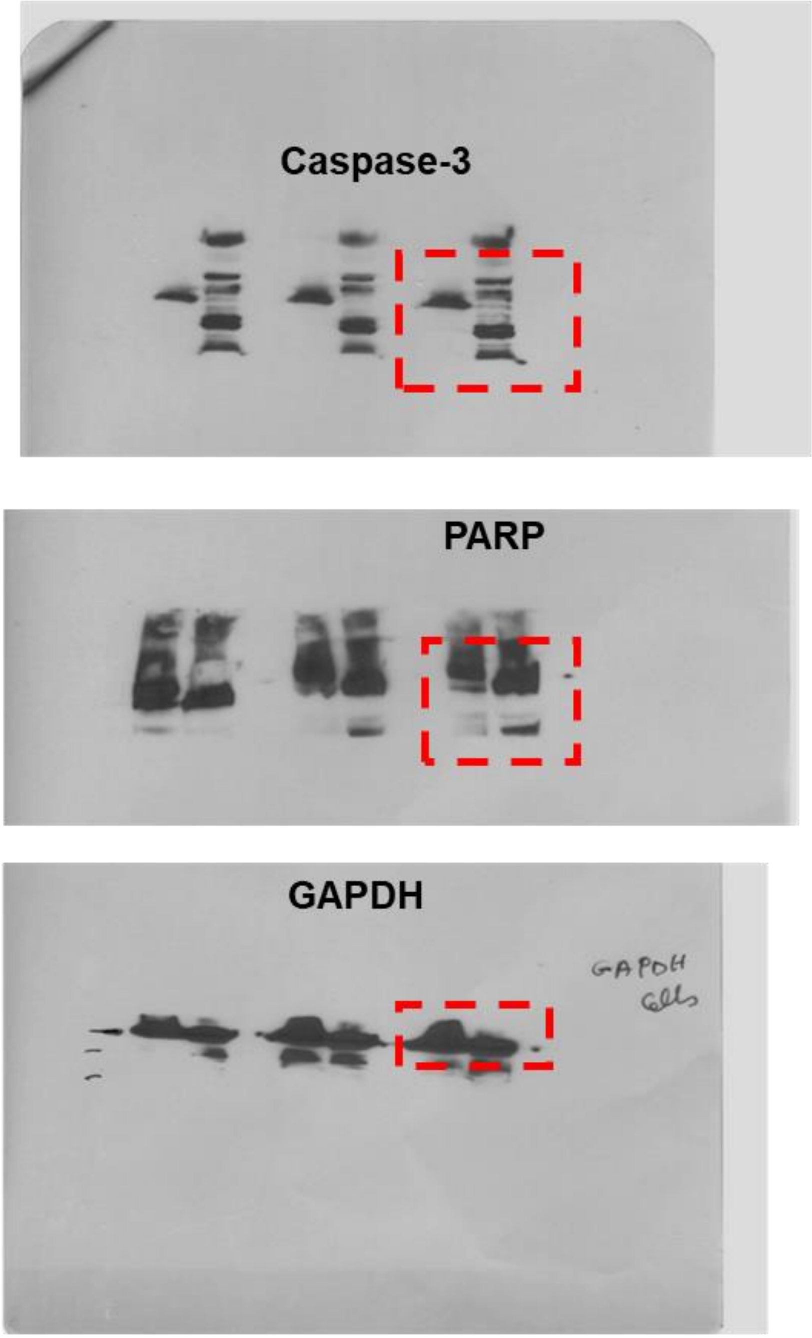

Figure 2E

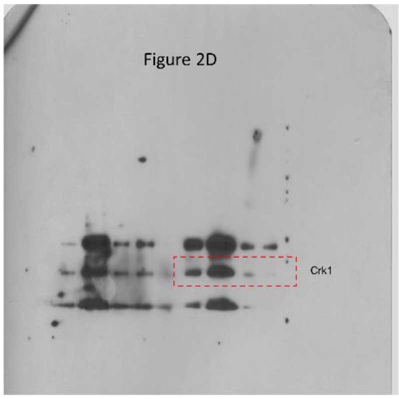

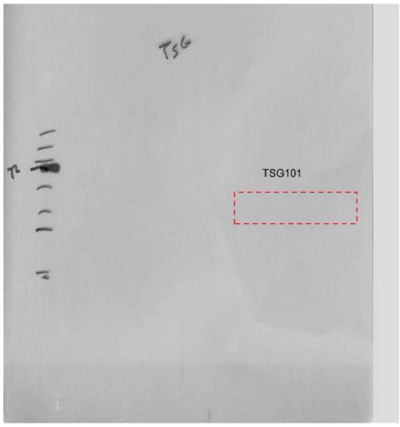

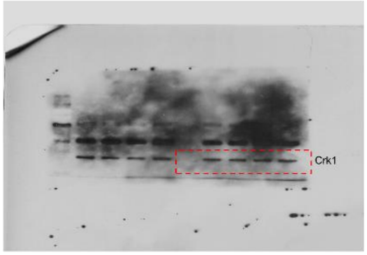

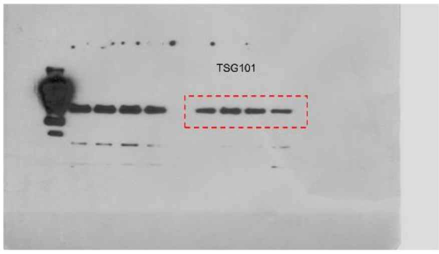

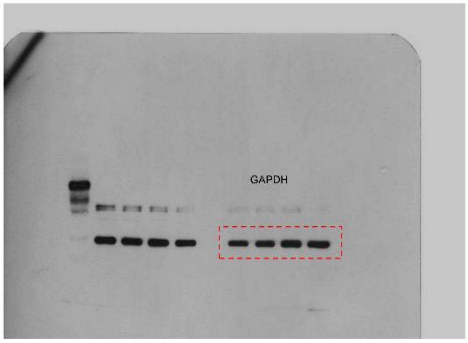

Figure 3A

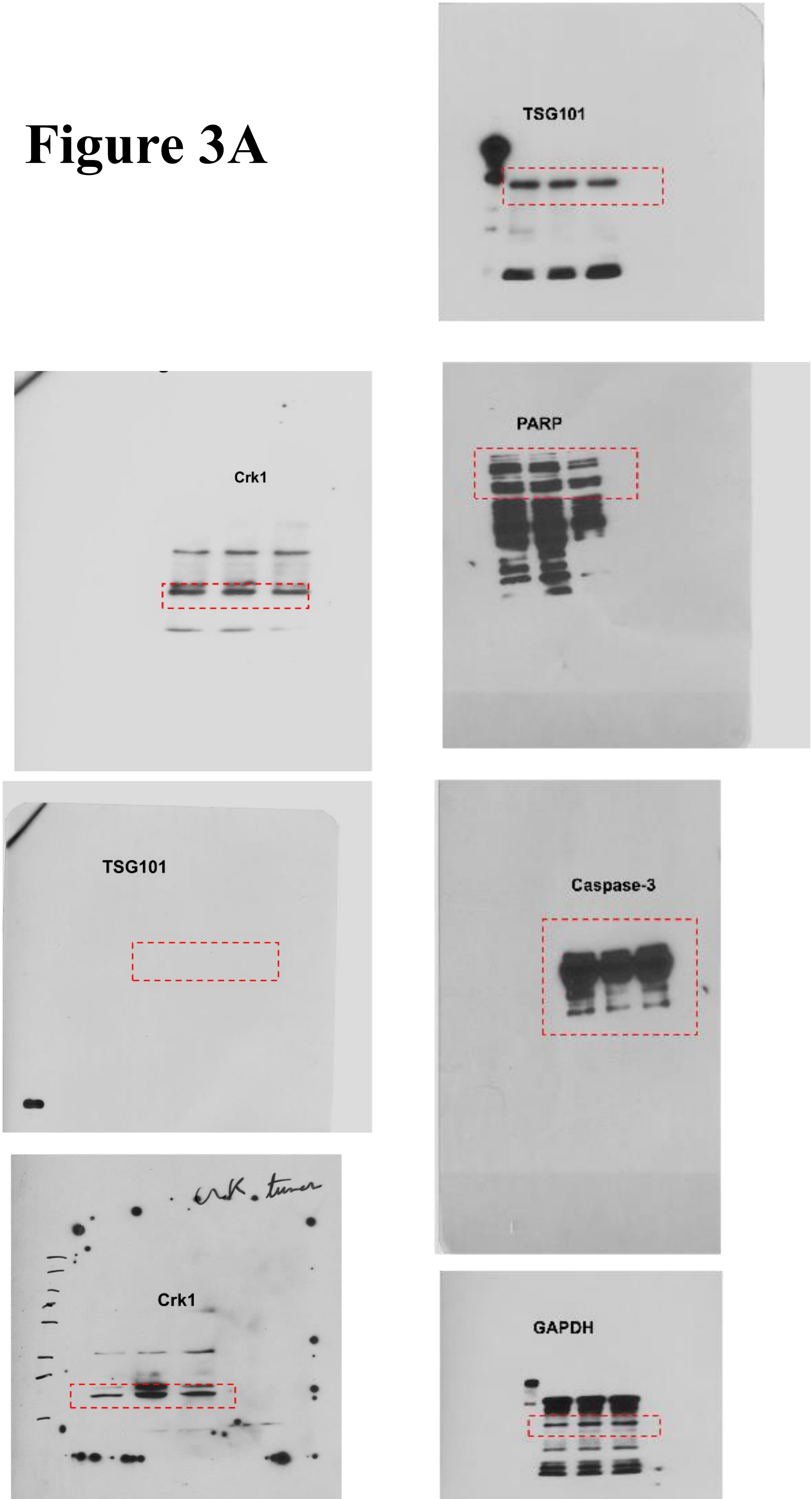

Supplement: Supplementary file 1 — Supplementary Figures. [file 41598_2022_8905_MOESM1_ESM.pdf]
